# Persistent free radicals in leaves as a stable standard for quantifying free radicals

**DOI:** 10.1016/j.mex.2025.103302

**Published:** 2025-04-03

**Authors:** Eric P. Vejerano, Khushboo Khushboo, Juan Vejerano

**Affiliations:** Department of Environmental Health Sciences, Arnold School of Public Health, University of South Carolina, Columbia, 29208, USA

**Keywords:** Biogenic persistent free radicals, BPFRs, Crape myrtle leaves, Electron paramagnetic resonance, ESR measurements, Organic radical quantification, DPPH alternative, Radical stability, Free radical calibration, Environmental applications, Alternative stable standard for radical measurement

## Abstract

This study explored plant‐derived biogenic persistent free radicals (BPFRs) in crape myrtle leaves as an alternative standard to 2,2‐diphenyl‐1‐picrylhydrazyl (DPPH) for quantifying organic radicals. Conventional methods rely on DPPH as a standard but are prone to degradation due to light, temperature, and humidity fluctuations. We performed electron spin resonance (ESR) measurements on both DPPH and leaf samples at various masses, temperatures (22 °C and 35 °C), and relative humidity (∼100 % RH) to evaluate radical stability. We observed consistent linear responses with increasing sample mass for crape myrtle leaves, similar to the behavior of DPPH. However, the BPFRs remained more stable under high temperature and humidity over seven days, retaining most of their radical signals compared to DPPH. The g‐factor of crape myrtle leaves remained nearly constant, indicating no significant alteration in the paramagnetic center. The peak‐to‐peak linewidth varied slightly, reflecting minor environmental and sample preparation differences. These findings suggest that BPFRs in plant tissue are more robust standards. Implementing leaf‐derived radicals as calibration references may enhance reproducibility in free radical quantification, reduce artifacts from DPPH degradation, and support broader environmental or biological applications.

• BPFRs in crape myrtle leaves exhibited excellent stability under elevated temperatures and humidity compared to DPPH, maintaining their radical signals over seven days.
• BPFRs demonstrated a consistent linear response with increasing sample mass, similar to DPPH, making them a viable alternative for free radical quantification.
• Using leaf-derived radicals as calibration standards may enhance reproducibility in free radical quantification and mitigate artifacts from the degradation of DPPH.

## Graphical abstract

**Figure:**
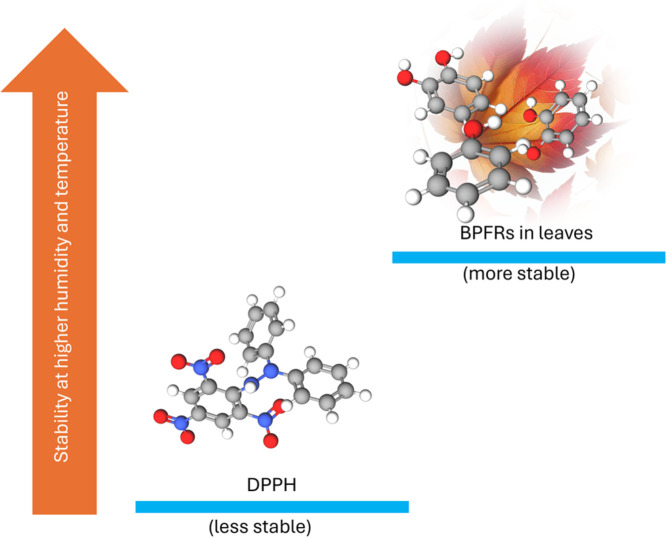

Specifications table

**Table:** 

Subject area:	Chemistry
More specific subject area:	Environmental chemistry
Name of your method:	Alternative stable standard for radical measurement
Name and reference of original method:	none
Resource availability:	Bruker EMX ESR spectrometer, silica 100–200 mesh, crape myrtle leaves, 2,2‐diphenyl‐1‐picrylhydrazyl (DPPH), and dichloromethane (purity 99.9 %), water (18 MΩ⋅cm).

## Background

Conventional approaches to quantifying free radicals using electron spin resonance spectroscopy (ESR) frequently rely on 2,2‐diphenyl‐1‐picrylhydrazyl (DPPH) [[1], [2], [3]] as a calibration standard because it is a stable radical. However, DPPH is sensitive to light, temperature, and oxygen [4], which can degrade its radical content and compromise data reproducibility for long-term radical measurement [[5], [6], [7]].

Recently, we have shown that leaves contain biogenic persistent free radicals (BPFRs) and are highly stable [8]. Following this result, we tested if they are better standards for ESR measurement due to their stability. Here, we compare the performance and stability of radicals in crape myrtle leaves against DPPH at different temperatures and at 100 % relative humidity level to assess their potential as a more stable standard for free radical quantification.

## Method details

### Materials and chemicals

Silica particles (100–200 mesh), DPPH, and dichloromethane (purity 99.9 %) were obtained from commercial suppliers and used without further purification. Nonsenescent crape myrtle (*Lagerstroemia speciosa*) leaves were collected from mature plants. All water used was deionized (18 MΩ⋅cm).

### Preparation of crape myrtle leaf samples

Nonsenescent crape myrtle leaves were washed thoroughly with deionized water to remove surface contaminants. The leaves were then air‐dried at room temperature (∼22 °C) for 48 h, followed by oven‐drying at 35 °C for three days to maximize moisture removal. Dried leaves were crushed using a food processor, further ground with a mortar and pestle, and sieved through a 250-µm mesh for uniform particle size. Precise amounts of this processed leaf material were weighed and mixed with silica particles to yield a total mass of 50 mg per sample.

### Preparation of DPPH samples

To prepare solid‐phase DPPH standards, 2.1 mg of DPPH was dissolved in dichloromethane. Subsequently, 2000 mg of silica was added, and the mixture was evaporated for 6 h, ensuring thorough drying. Aliquots of this dried material (5–25 mg) were then weighed out, and silica was added to achieve a final mass of 50 mg per sample. All samples were packed to the same height to ensure consistent packing density. These samples served as comparison standards for all ESR measurements.

### Radical stability tests

*Temperature exposure (22* °*C and 35* °*C)***.** Samples were stored at either room temperature (22 °C) or elevated temperature (35 °C) in sealed containers. ESR signal intensities were measured at Day 0, Day 1, and Day 7 to assess how each radical source (DPPH vs. BPFRs) responded to temperature over time.

*Relative humidity exposure (∼100 % RH)***.** Samples were placed in a controlled humidity chamber maintained at ∼100 % relative humidity. ESR measurements were again performed on Day 0, Day 1, and Day 7 to evaluate radical stability under prolonged moisture exposure.

## Method validation

### ESR measurement and method validation

All samples were placed in standard quartz ESR tubes. The instrument parameters (e.g., microwave power, modulation amplitude, and sweep width) were optimized to avoid signal saturation and ensure reproducibility. We acquired the spectra using an X-band Bruker EMXplus ESR spectrometer, 100 kHz, and microwave frequency 9.53 GHz. All spectra were obtained at room temperature using these parameters: microwave power 1 mW, modulation amplitude 4 G, sweep width 200 G, time constant 40.960 ms, and sweep time 167.7 s, using a receiver gain of 1.0 × 10^4^. Double integration of the first‐derivative ESR signal was used to calculate radical concentration.

### Mass‐dependent signal and spectral comparisons

**Fig. 1:**
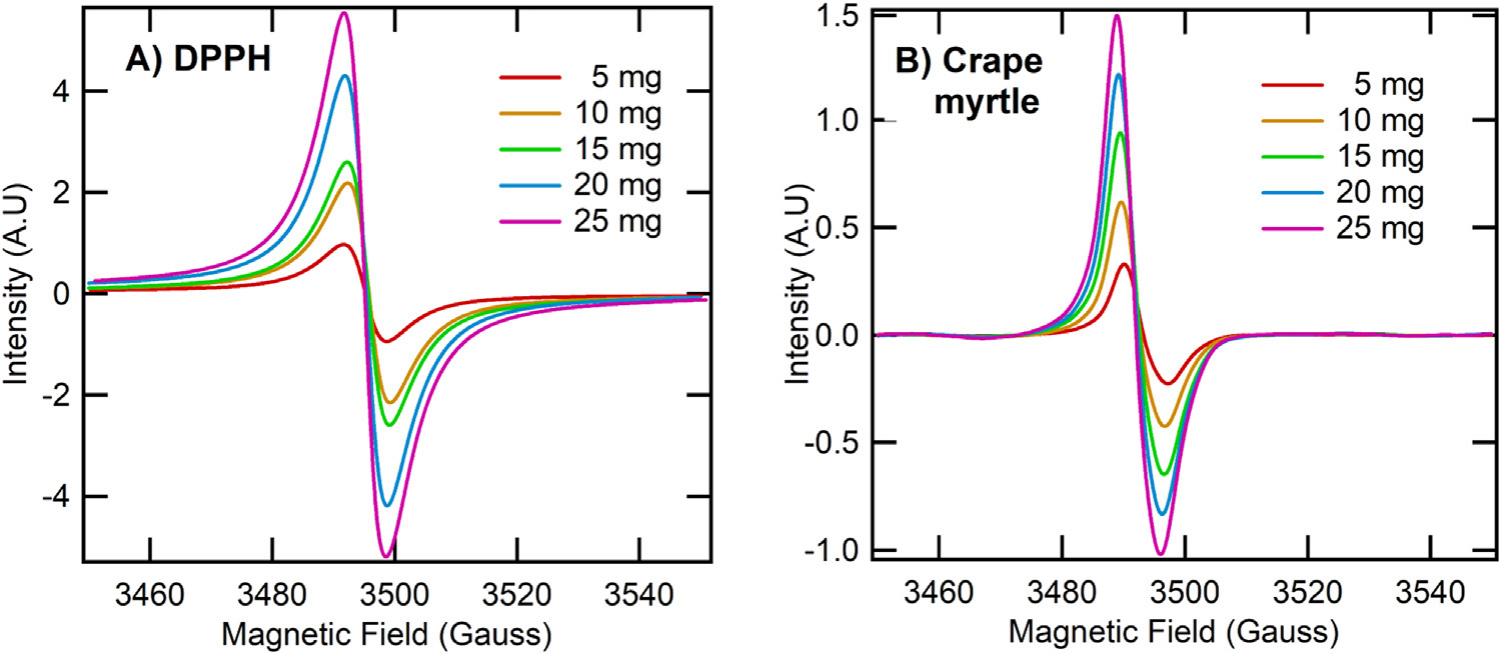
First-derivative electron spin resonance (ESR) spectra of DPPH (A) and crape myrtle (B) at varying sample concentrations or leaf masses.

Representative first‐derivative ESR spectra of DPPH and crape myrtle leaf samples at increasing masses exhibited a proportional rise in signal intensity with sample mass (Fig. 1). However, DPPH displayed a higher overall intensity due to its purity. The ESR spectra for crape myrtle have a much narrower linewidth than that of DPPH and less baseline drift. This attribute enables the integration interval to be clearly delineated within the magnetic field range (3450–3550 G) commonly used for scanning organic radicals, resulting in a more precise determination of the area and, thus, the concentration.

### Calibration curves and normalized responses

**Fig. 2:**
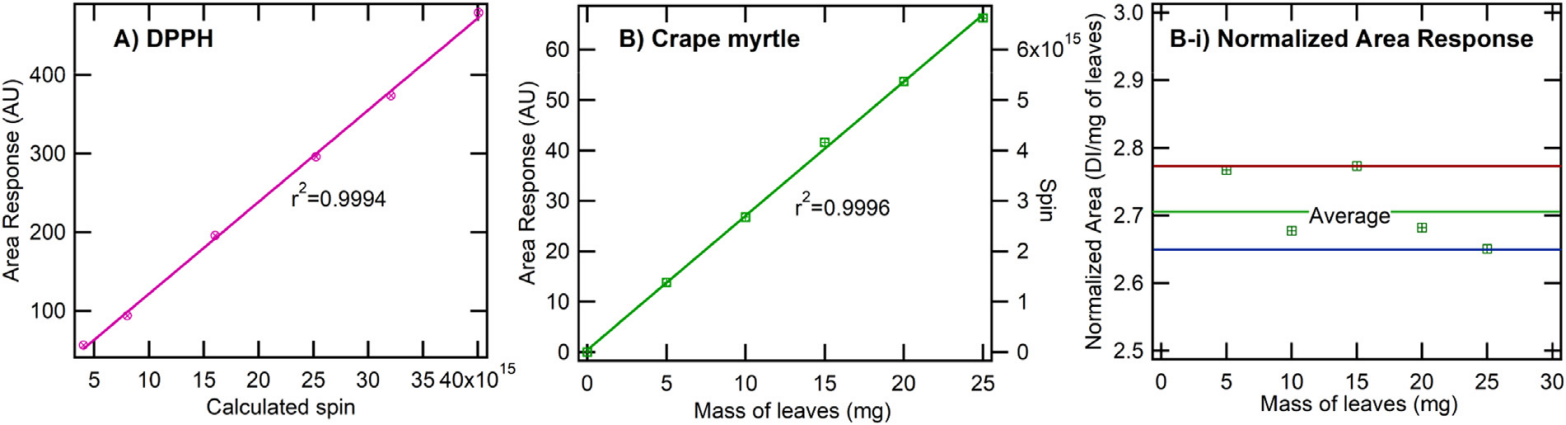
Calibration curves for ESR measurements. (A) Linear correlation between area response (in arbitrary units, AU) and the number of DPPH spins, showing a strong linear relationship. B) Relationship between the area response and the mass of crape myrtle leaves (mg), with the corresponding spin count indicated on the secondary (right) axis. (B-i) The normalized area response for crape myrtle.

*DPPH Calibration.* Plotting the integrated ESR signal (AU) against calculated spins of DPPH yielded an exceptional linear fit (R² = 0.9996) (Fig. 2A). Fig. 2 presents calibration curves and corresponding analyses for the ESR measurements. In Fig. 2A, a standard curve was established using DPPH as a radical reference. The linear correlation between the integrated ESR signal (area response, in arbitrary units) and the total number of DPPH spins was very strong (r² = 0.9994). We then compared this calibration standard to that of crape myrtle.

*Crape myrtle calibration*. Earlier, we established that an increasing leaf mass loading increases the ESR signal of BPFRs [8]. Still, the BPFR distribution's uniformity and adequacy for generating a calibration curve remains uncertain. To assess linearity, we tested leaf samples across a range of masses (Fig. 2B). The integrated ESR signal increased linearly with increasing leaf mass, with an r² = 0.9996, which is higher than that obtained for DPPH. The linearity of these data indicates that the concentration of radical species (or at least the ESR-active species) in crape myrtle leaves is proportional to the sample mass over this tested range. The strong linear relationship between leaf mass and ESR signal (Fig. 2B) confirms that the concentration of ESR-active species scales linearly with the amount of plant material. The high degree of linearity implies no substantial sample-matrix effects, such as signal quenching or nonlinear absorption, over the examined mass range.

*Normalized area*. Fig. 2B-i, the ESR signal was normalized by dividing the integrated area by the corresponding leaf mass, yielding the normalized area response. Normalizing the ESR area to leaf mass produced a consistent value (∼2.7 AU mg⁻¹), with data points deviating by less than ±2 % (Figure 2B‐i). Such tight clustering demonstrates the measurement's reproducibility and suggests that plant‐derived radicals are uniformly present per unit mass of the dried powdered leaves. The near-constant normalized area indicates that the BPFR spin density remains relatively uniform over the mass range tested.

### Consistency of g‐factor and linewidth

**Fig. 3:**
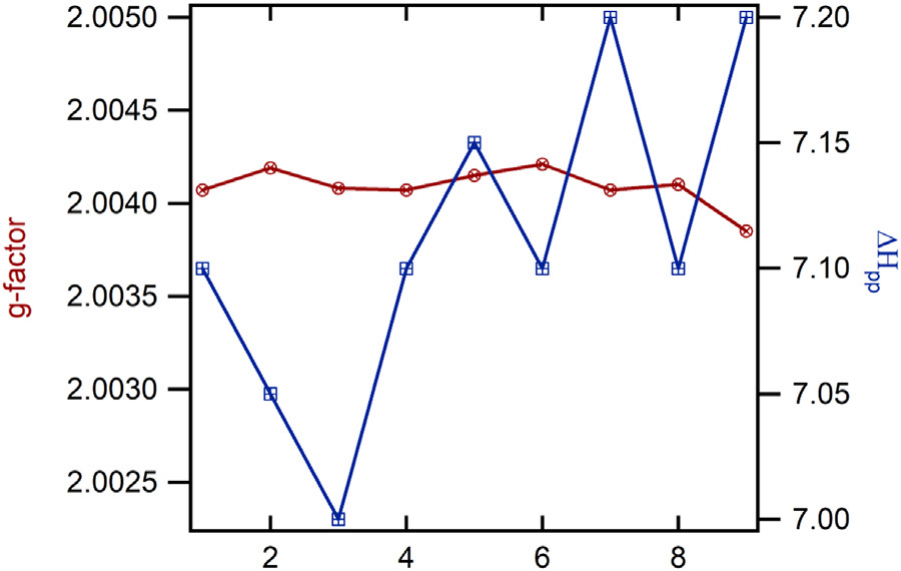
Variation in ESR properties (g-factor and peak-to-peak width (ΔH_pp_)) of crape myrtle pooled from different measurements (varying time, RH, and temperature).

Fig. 3 shows the variation in ESR properties of crape myrtle pooled from measurements at different conditions. Measurements of the g‐factor (red circles) revealed stable values around 2.0040, indicating that the electronic structure of the BPFRs remained effectively unchanged across multiple samples and conditions (i.e., varying time, RH exposure, and temperature). The peak‐to‐peak linewidth (ΔH_pp_, blue squares) varied modestly between 7.00 and 7.20 G. This slight variation is likely due to minor differences in sample packing or local environments (e.g., slight variations in moisture) rather than to any fundamental shift in the properties of BPFR species. These results show that the BPFR paramagnetic centers in leaves are highly stable under different conditions.

### Effects of relative humidity on ESR spectra

**Fig. 4:**
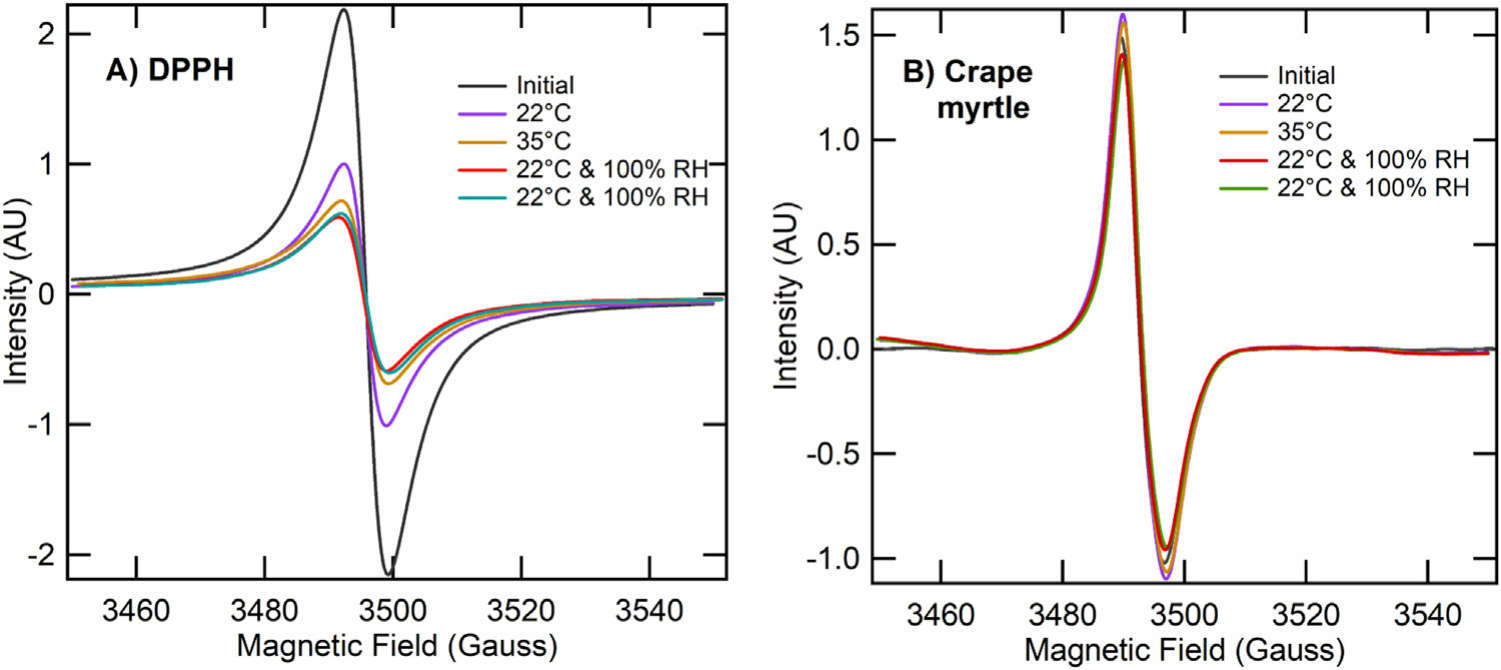
Response of ESR signal with temperature (22 and 35 °C**)** and relative humidity for DPPH and crape myrtle.

When subjected to 100 % relative humidity level, DPPH (Fig. 4A) retained nearly identical ESR signals regardless of moisture, confirming its short‐term stability. Crape myrtle leaves (Figure 4B) likewise showed minimal signal position or shape changes, suggesting that elevated relative humidity over brief periods did not substantially quench their paramagnetic centers. These short‐term humidity tests show that BPFRs in leaves remain extremely stable under saturated relative humidity.

### Stability over time under varying conditions

**Fig. 5:**
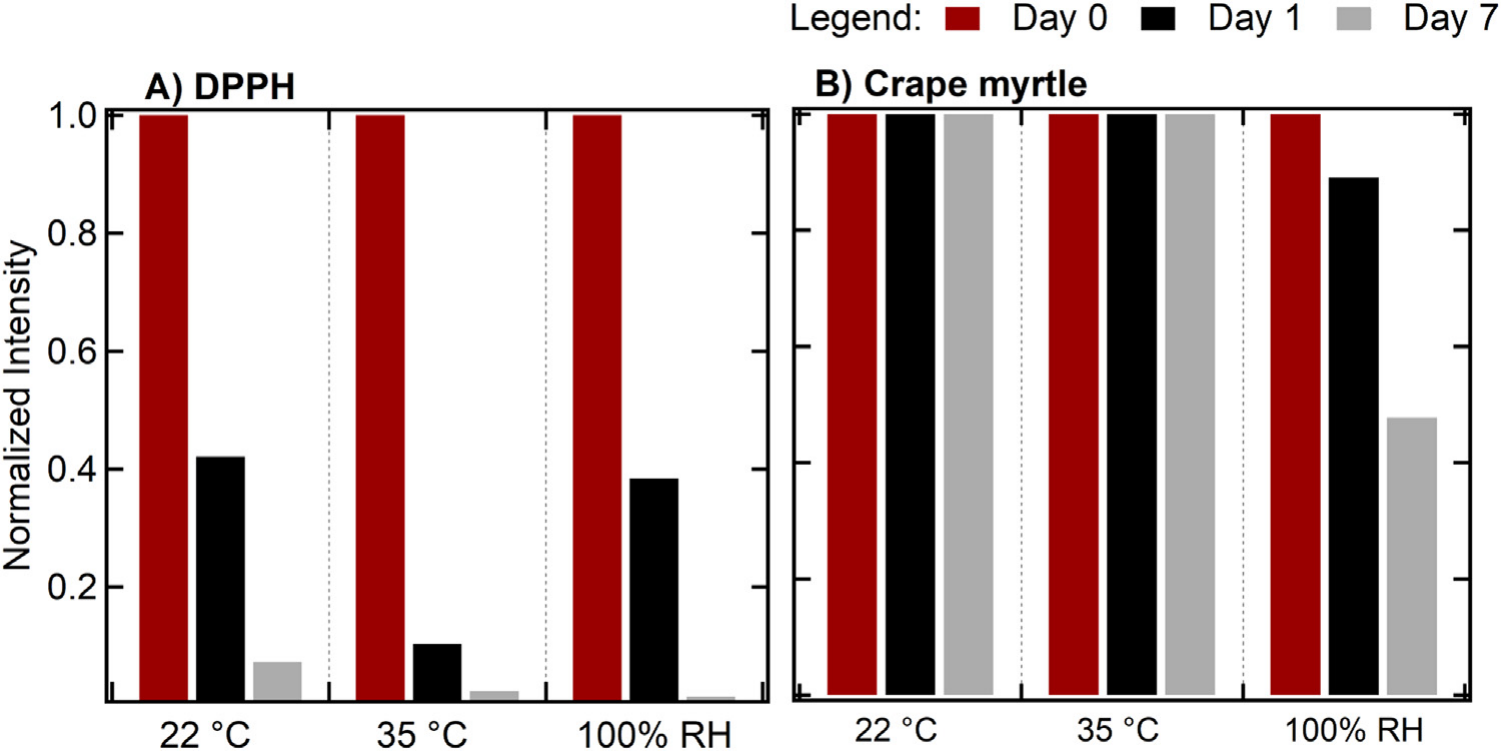
Radical signals from (A) DPPH and (B) crape myrtle at 22 °C, 35 °C, and RH conditions across days 0, 1, and 7 days.

*DPPH standard*. At 22 °C, the normalized radical intensity gradually diminished by Day 7 (Fig. 5A). A more significant decrease was observed at 35 °C, indicating that higher temperatures accelerate DPPH radical degradation or structural reorganization. Under 100 % RH, there was an initial drop on Day 1 similar to that under 22 °C and almost a non-detectable signal on Day 7.

*Crape myrtle leaves***.** BPFRs appeared notably more stable at 22 °C and 35 °C, with almost no decline in ESR intensity for over a week (Fig. 5B). Although 100 % RH exposure led to a moderate reduction in the radical signal by Day 7, the decrease was less severe than that seen for DPPH at 22 °C, 35 °C, and at similar relative humidity. These observations imply that BPFRs can better resist temperature‐ and relative humidity‐induced degradation over the test periods studied.

### Comparative stability and implications for standardization

These results demonstrate that leaf‐derived BPFRs are a more robust standard for quantifying free radicals. While DPPH remains a widely recognized reference, our data shows that crape myrtle leaves exhibit more consistent signals under various temperature and humidity conditions. Compared to synthetic standards like DPPH, BPFRs offer notable advantages, including enhanced stability and cost-effectiveness.

We expect leaves from other plants to follow the same behavior since we observed them to have similar characteristics in our earlier studies [8]. The linear response with mass, tight clustering of normalized intensities, and minimal changes in the physico-chemical properties (inferred from g‐factor stability and ΔH_pp_) demonstrate that BPFRs are stable calibration standards.

Although BPFR content varies across different growth stages of plants (i.e., higher in senescent than non-senescent leaves), both exhibit the same stability [8]. Thus, variation in free radical content does not compromise their use as a quantification standard, provided that leaves from a consistent growth stage are used. Additionally, the variable g-factors of BPFRs from different plant species preclude their use for calibrating instability in the ESR spectrometer's center field unless a consistently processed leaf batch from the same species is used throughout the experiment to ensure uniform radical properties. BPFRs are highly suitable for quantifying free radical concentrations and conducting long-term kinetic studies.

This enhanced stability is especially appropriate for studies conducted in non‐ideal or variable experimental environments, where fluctuations in temperature and moisture can compromise traditional synthetic radical standards like DPPH. This alternative standard is crucial for the long-term measurement of free radical signals, as it eliminates the need for repeated DPPH preparation, which is tedious and prone to variability. Protocols should include controlled drying conditions, ensuring particle size uniformity by sieving, and using processed leaves from the same species and batches, which are crucial for standardization and reproducibility. These findings provide simple to prepare and more reliable alternative standards for quantifying free radicals using ESR spectrometers.

## Limitations

None.

## Ethics statements

None.

## CRediT author statement

**Eric P. Vejerano:** conceptualization, sample preparation, data presentation, analysis, writing, editing, reviewing, project administration and supervision. **Khushboo Khushboo:** ESR measurement and data analysis, writing draft, experimentation. **Juan Vejerano:** sample preparation, experimentation, data analysis, writing draft, data presentation, and experimentation.

## Supplementary material *and/or* additional information

None.

## Declaration of competing interest

The authors declare that they have no known competing financial interests or personal relationships that could have appeared to influence the work reported in this paper.

## Data Availability

Data will be made available on request.
